# Osteoporosis screening using machine learning and electromagnetic waves

**DOI:** 10.1038/s41598-023-40104-w

**Published:** 2023-08-08

**Authors:** Gabriela A. Albuquerque, Dionísio D. A. Carvalho, Agnaldo S. Cruz, João P. Q. Santos, Guilherme M. Machado, Ignácio S. Gendriz, Felipe R. S. Fernandes, Ingridy M. P. Barbalho, Marquiony M. Santos, César A. D. Teixeira, Jorge M. O. Henriques, Paulo Gil, Adrião D. D. Neto, Antonio L. P. S. Campos, Josivan G. Lima, Jailton C. Paiva, Antonio H. F. Morais, Thaisa Santos Lima, Ricardo A. M. Valentim

**Affiliations:** 1 Laboratory of Technological Innovation in Health (LAIS), Natal, RN Brazil; 2 Advanced Nucleus of Technological Innovation (NAVI), Federal Institute of Rio Grande do Norte (IFRN), Natal, RN Brazil; 3 LyRIDS, ECE-Engineering School, Paris, France; 4 Department of Informatics Engineering, Univ. Coimbra, Centre for Informatics and Systems of the University of Coimbra (CISUC), Coimbra, Portugal; 5 Department of Electrical and Computer Engineering, School of Science and Technology, New University of Lisbon, Lisbon, Portugal; 6 Post-Graduation Program on Electrical and Computer Engineering, Federal University of Rio Grande do Norte, Natal, RN Brazil; 7 University Hospital Onofre Lopes, Federal University of Rio Grande do Norte (UFRN), Natal, RN Brazil; 8 Ministry of Health, Brasília, Brazil

**Keywords:** Machine learning, Biomedical engineering

## Abstract

Osteoporosis is a disease characterized by impairment of bone microarchitecture that causes high socioeconomic impacts in the world because of fractures and hospitalizations. Although dual-energy X-ray absorptiometry (DXA) is the gold standard for diagnosing the disease, access to DXA in developing countries is still limited due to its high cost, being present only in specialized hospitals. In this paper, we analyze the performance of Osseus, a low-cost portable device based on electromagnetic waves that measures the attenuation of the signal that crosses the medial phalanx of a patient’s middle finger and was developed for osteoporosis screening. The analysis is carried out by predicting changes in bone mineral density using Osseus measurements and additional common risk factors used as input features to a set of supervised classification models, while the results from DXA are taken as target (real) values during the training of the machine learning algorithms. The dataset consisted of 505 patients who underwent osteoporosis screening with both devices (DXA and Osseus), of whom 21.8% were healthy and 78.2% had low bone mineral density or osteoporosis. A cross-validation with k-fold = 5 was considered in model training, while 20% of the whole dataset was used for testing. The obtained performance of the best model (Random Forest) presented a sensitivity of 0.853, a specificity of 0.879, and an F1 of 0.859. Since the Random Forest (RF) algorithm allows some interpretability of its results (through the impurity check), we were able to identify the most important variables in the classification of osteoporosis. The results showed that the most important variables were age, body mass index, and the signal attenuation provided by Osseus. The RF model, when used together with Osseus measurements, is effective in screening patients and facilitates the early diagnosis of osteoporosis. The main advantages of such early screening are the reduction of costs associated with exams, surgeries, treatments, and hospitalizations, as well as improved quality of life for patients.

## Introduction

Osteoporosis is characterized by impaired bone strength^1^ and affects approximately 6.3% of men over the age of 50 and 21.2% of women over the same age range globally, i.e., approximately 500 million men and women worldwide^2^. The disease causes more than 8.9 million fractures annually, resulting in one osteoporosis fracture every 3 s^3^. In the US, the annual direct medical cost of osteoporosis in 2005 was 17 billion USD and is projected to rise to 25 billion USD by 2025^4^. In the 27 countries of the European Union, the estimated cost was 34.5 billion dollars in 2010. In four latin american countries (Brazil, Mexico, Colombia, and Argentina), the burden of the disease in 2018 was estimated at 1.17 billion dollars^5^. In Brazil, a 63% increase in the annual number of fractures is estimated by 2030 compared to 2015, in individuals aged between 50 and 89 years. In addition, 57–60% of the population at high risk for osteoporosis does not receive any treatment^6^.

The most common exam to diagnose osteoporosis is the measurement of bone mineral density (BMD) through dual-energy X-ray absorptiometry (DXA)^2^ reported in g/cm^2^. Through the DXA exam applied to the lumbar spine, femur, and forearm, the World Health Organization (WHO) defines the international reference, establishing 3 categories based on a T-score index. For postmenopausal women and men over 50, a T-score of − 1.0SD or greater represents normal conditions, from − 1.0 to − 2.5 SD low BMD and − 2.5 SD or less for osteoporosis. The disease cannot be diagnosed for people under 50 years old based only on BMD, therefore, a Z-score normalization is recommended. In this situation a score inferior to − 2.0 SD indicates low BMD and above − 2.0 SD indicates normality^7^. However, DXA requires a room with an infrastructure that can handle the equipment, which is large, expensive and requires specialized professionals to operate it.

Identifying the risk factors of the disease in its initial stage and referring it to specialized care gives more chances for a better result in the treatment and prognosis of the cases^8^. In addition, the qualification of diagnosis at this level of care is important, as it promotes equity in the system, reducing the waiting list for access to specialized care, which can improve the entire regulatory process. Early diagnosis of osteoporosis enables the prevention of osteoporotic fractures and a lower cost to the public health system, since treatments are more effective in the early stages of the disease, before the appearance of fractures, to save expenses with surgeries and hospitalizations^9^. Some studies have used algorithms to screen and make appropriate referrals for further testing in patients with suspected osteoporosis. Machine Learning (ML) algorithms were used to compute the risk and identify the factors linked to such risk of osteoporosis and low BMD^10^. One of these papers studied rheumatic patients in China, and the authors showed, based on ML techniques, that age and body mass index were the most important factors for low BMD in the lumbar spine and femur^11^. However, most systems based on ML techniques, which are being widely developed for pattern recognition and BMD value estimation, still require medical imaging (which is usually associated with DXA or X-Ray)^12^.

Osseus is a portable and low-cost device with easy access to the population that is non-invasive and can assist in the classification of bone mineral density. Osseus works by reading a radiofrequency signal at 2.44 GHz^13^. This microwave signal passes through the medial phalanx of the patient's middle finger and is received by the Osseus. Some papers demonstrated that the phalanx region is a useful site for bone measurements, as it is surrounded by small amounts of soft tissue, since large amounts of soft tissue around the bone reduce the accuracy of the measurement^14^. In addition, they are located in the hands where there are no internal organs prone to the effects of radiation, resulting in a reduction in the effective dose for x-ray based examinations^15^ like the Schick AccuDEXA Portable Bone Densitometer^16^, that measures the bone mineral density in the middle phalanges of the third finger. The phalanges have anatomical and structural peculiarities such as the parallelism between the lateral faces, facilitating the application of emitting and receiving transducers^17^. VENDIK and collaborators^18^ demonstrated in their study that the dielectric constants for healthy and diseased bones are significantly different, making it possible to diagnose osteoporosis through the development of an appropriate measurement procedure in the phalangeal region. For this reason, the Osseus device uses the middle phalanx region of the middle finger. The Osseus processes the signal along with other patient's health features (Supplementary Table 1), outputting a prognosis for the possible need to subsequently perform a DXA exam. We integrate ‘Osseus results’ with several patient characteristics such as age and sex. Using supervised classification algorithms, we train ML models to predict changes in BMD.

## Results

505 patients with a mean age of 62.2 years (range 18–101 years) were recruited between July 2021 and June 2022 at the University Hospital Onofre Lopes of Federal University of Rio Grande do Norte (HUOL/UFRN) under the ethics committee CAAE-No. 39675020.0.0000.5292/2020. Among these patients, 110 (21.8%) were healthy, and 395 (78.2%) had low BMD or osteoporosis. The patients' characteristics are presented in Table 1.

**Table 1:** Demographic characteristics of the sample.

Characteristics	Patients (N = 505), n (%)	Men (N = 46), n (%)	Women (N = 459), n (%)
Age < 50 years	71 (14,1)	13 (2,6)	58 (11,4)
Age ≥ 50 years	434 (85,9)	33 (6,5)	401 (79,4)
Alcohol	63 (12,5)	7 (1,4)	56 (11,1)
Smoking	187 (37,0)	15 (8,0)	172 (34,0)
Physical activity	121 (24,0)	11 (2,2)	110 (21,8)
Milk Intake	402 (79,6)	31 (6,1)	371 (73,5)
Calcium intake	205 (40,6)	13 (2,6)	192 (38,0)
Vitamin D intake	211 (41,8)	14 (2,8)	197 (39,0)
Fracture history	94 (18,6)	9 (1,8)	85 (16,8)
Osteoporosis family history	170 (33,7)	11 (2,2)	159 (31,5)
Curved parents	17 (3,4)	0 (0,0)	17 (3,4)
Corticosteroids	135 (26,7)	20 (4,0)	115 (22,7)
Rheumatoid arthritis	42 (8,3)	1 (0,2)	41 (8,1)
Diseases (Hyperthyroidism or diabetes)	235 (46,5)	22 (4,4)	213 (42,1)
Menopause	411 (81,4)	–	411(81,4)

As the patients were already referred to undergo DXA, the number of men and women aged over 50 was 434 (85.9%), of which 401 (79.4%) were postmenopausal women. The total number of male subjects was 46 (9.1%).

### Model performance

The comparative results of the performance of the 19 supervised classifiers are displayed in descending order of F1 (Table 2). The positive class corresponds to the patient who has low bone mineral density, or osteoporosis. The negative class corresponds to a healthy patient.

**Table 2:** Classification reports of all classifiers.

Classification algorithm	TNR	FPR	FNR	TPR	NPV	PPV	F1	Accuracy	AUC
Random forest	0.9104	0.0896	0.1219	0.8781	0.8794	0.9106	0.8937	0.8940	0.8943
XG boost	0.9009	0.0991	0.1500	0.8500	0.8546	0.8994	0.8734	0.8751	0.8754
Gradient boosting	0.8720	0.1280	0.1344	0.8656	0.8656	0.8753	0.8694	0.8687	0.8688
Extra trees	0.8848	0.1152	0.1469	0.8531	0.8550	0.8842	0.8681	0.8688	0.8690
Hist grad boosting	0.8849	0.1151	0.1719	0.8281	0.8357	0.8837	0.8535	0.8561	0.8565
Bagging	0.9041	0.0959	0.2031	0.7969	0.8131	0.8972	0.8431	0.8497	0.8505
Ada boost	0.8432	0.1568	0.1625	0.8375	0.8357	0.8505	0.8421	0.8402	0.8404
Gaussian process	0.9487	0.0513	0.2469	0.7531	0.7923	0.9394	0.8340	0.8497	0.8509
XG boost RF	0.8401	0.1599	0.1781	0.8219	0.8218	0.8441	0.8315	0.8308	0.8310
Decision tree	0.8591	0.1409	0.2125	0.7875	0.7973	0.8552	0.8189	0.8228	0.8233
Linear discriminant	0.7823	0.2177	0.1719	0.8281	0.8162	0.7973	0.8119	0.8055	0.8052
Logistic regression	0.7919	0.2081	0.1844	0.8156	0.8078	0.8014	0.8081	0.8039	0.8037
K neighbors	0.8911	0.1089	0.2906	0.7094	0.7499	0.8716	0.7813	0.7991	0.8002
Linear SVC	0.6486	0.3514	0.1625	0.8375	0.8278	0.7428	0.7719	0.7439	0.7430
Quadratic discrimant	0.8591	0.1409	0.2875	0.7125	0.7448	0.8382	0.7701	0.7849	0.7858
SVC	0.7374	0.2626	0.2313	0.7688	0.7581	0.7514	0.7589	0.7532	0.7531
Stochastic gradient desc	0.8300	0.1700	0.3219	0.6781	0.7206	0.8060	0.7329	0.7532	0.7541
Extra tree	0.8045	0.1955	0.3156	0.6844	0.7129	0.7854	0.7303	0.7437	0.7444
Gaussian naive bayes	0.8463	0.1537	0.3469	0.6531	0.7040	0.8150	0.7246	0.7485	0.7497

*TNR* True Negative Rate, *FPR* False Positive Rate, *FNR* False Negative Rate, *TPR* True Positive Rate, *NPV* Negative Predictive Value, *AUC* Area Under the Curve.

**Figure 1:**
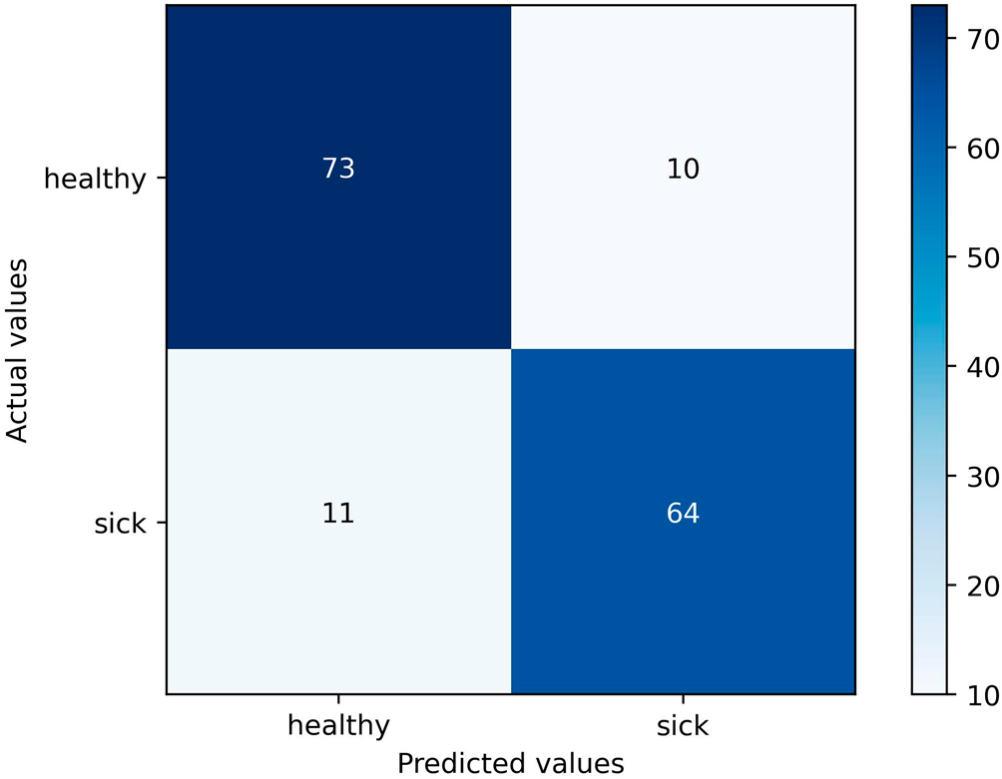
Confusion matrix for the final model.

Since the RF method achieved the best performance in terms of F1 (0.8937), accuracy (0.8940) and AUC (0.8943), we present the RF model results using the test portion of the dataset. The original study sample contains 505 patients, from these 110 belong to the healthy class and 395 belong to the sick class (low bone mineral density and osteoporosis). The SMOTE method resampled the healthy class by creating synthetic examples based on the real ones. So the healthy class examples passed from 110 to 395 samples which generated a final balanced dataset containing 790 samples (50% healthy and 50% sick). This final dataset is then used to generate the results. Using the RF model to the final dataset, in the confusion matrix (Fig. 1) can be observed that in a sample of 158 patients (20% of 790), with 83 patients in a healthy condition, the model correctly indicated the health condition of 73 (87,95%) as healthy (specificity) and recommended that 10 (12,05%) took the DXA test when they didn't need it (false positive rate). Out of the 75 sick patients, the model erroneously indicated the condition of 11 (14,67%) patients as healthy (false negative rate) and correctly recommended that 64 (85,33%) undergo the DXA exam (recall). Out of the 84 predicted healthy, the model hit 86,90% (Negative Predictive Value), and of the 74 predicted sick, the model hit 86,49% (positive predictive value). The accuracy obtained in the tests was 86,71% and F1-score = 0.8591.

**Figure 2:**
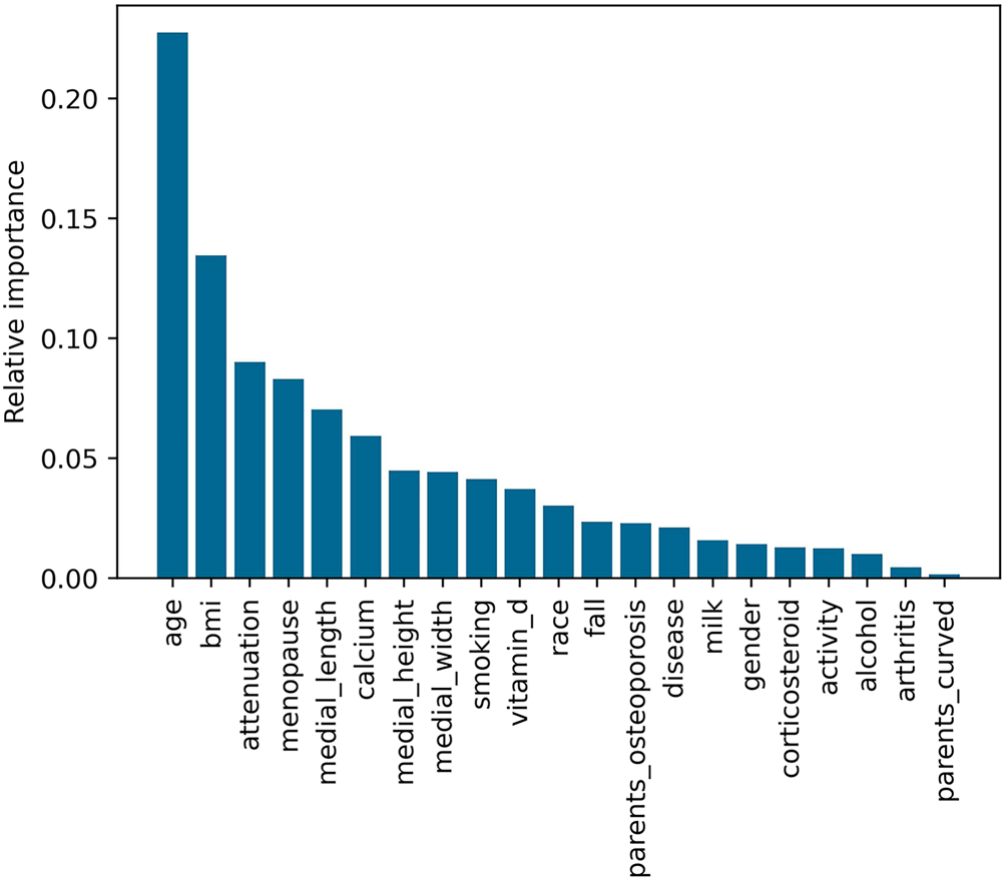
Importance of variables for the final classification of the model.

In the RF analysis, it was possible to visualize the importance of the variables in the final result (Fig. 2) based on the degree of impurity of the tree nodes. The less impure the division is after going through this feature, the more important the feature. Age, BMI (weight/height^2^) and the signal's attenuation (Osseus result) were the most important variables to split the data into the two classes (healthy and sick). The individual features’ importance is described in the supplementary table 2.

Combining the Osseus attenuation signal with the set of presented features the method takes into account an important feature that gives an index of bone porosity through the microwave attenuation signal. All the features together give a meaningful result. For more details about the individual features’ importance please refer to supplementary Table 2. There are no statistically significant differences if we compare the accuracy results when using the Osseus attenuation signal to the accuracy results without the Osseus signal. Even though we have better accuracy when using Osseus, this gain is not statistically significant. However, when we compare the accuracy results by taking out any other individual feature we do not find statistically significant differences either (Comparison all features vs. without Osseus T-test: 0.75, p-value: 0.46). If we compare the accuracy results of the test without the age (the most important feature) against the ones of the test that uses all features we will also find no statistically significant differences (Comparison all features vs. without age T-test: 1.70, p-value: 0.10). There is no individual feature that is capable of significantly changing the accuracy results in this study. The RF needs to rely on the set of features to correctly classify the patients, something like the rationale a doctor uses to make a decision. The doctor asks for a set of clinical exams before being able to affirm whether the patient is sick or healthy. What we can affirm in our study is that by combining the Osseus attenuation signal with the set of presented features the method takes into account an important feature that gives an index of bone porosity through the microwave attenuation signal. For more details see Supplementary Figs. 1a, b, c, d.

## Discussion

Computational methods based on Machine Learning are gaining a lot of prominence in healthcare applications. One of the main reasons for using machine learning in health care is due to the flexibility and scalability of these methods. Unlike traditional statistical methods that rely on inferring relationships between variables, machine learning is able to make predictions about the status of a patient (e.g., sick or healthy) based on other information from that patient^19^.

This process resembles the logic used by a doctor who orders tests to judge whether a patient is sick or not. To perform this judgment the machine learning algorithm is trained with data from several patients where the health status is known, and from this the method learns the patterns found in the data to separate each patient in each class. Once trained, the method is able to judge patients whose current status is unknown (sick or healthy) from their test results and personal profile.

As demonstrated in our study, this computational method was applied in the classification of osteoporosis cases and showed a good accuracy. However, our objective is not to obtain a final diagnosis about the cases but to help the doctor in the triage of the cases that will need a more specific and specialized exam, as for example, the DXA. The final decision will always be the doctor's, but our method can help in this decision making. The results showed that, in addition to age and BMI being the most important variables for the classification of osteoporosis^11^, the percentage of Osseus attenuation was the third most important variable. The selection of a subset of features based on their ranked importance could contribute to improving the model's performance in future work.

A point that must be evaluated in the real database, which was unbalanced, is that 110 patients (21.8%) had a healthy result, that is, they did not need to undergo the test. Reducing the number of healthy patients awaiting a DXA scan is one of the goals of Osseus. In this context, Osseus technology is a point of care digital health solution based on Machine Learning that acts as a tool to better qualify the screening of patients who will really need a DEXA exam. Therefore, when applied to primary health care through telehealth, it allows physicians to remotely access triage data (Osseus data) and more equitably regulate patients for the specialized healthcare. Regarding the ML model, it means reducing false positives. On the other hand, reducing false negatives is extremely important since patients who are really sick must not fail the DXA screening. So, the most important metric for this application was the F1, which seeks to maximize sensitivity and precision at the same time since it is a harmonic mean of both metrics while at the same time reducing false positive and false negative rates.

The metrics obtained for the RF model were similar to those found by Kerketta^20^ in which the Random Forest classifier was considered the best when compared to the decision tree and KNNeighbor (KNN) and was able to clearly identify the different stages of loss of bone mass density in the presence of tissue variations by computer simulation^20^. Our results reinforce the previous results of this algorithm's performance in the prediction of osteoporosis.

Most studies related to the use of ML in the diagnosis of osteoporosis use imaging tests to compose the most important predictive variables of the algorithm, whether using radiographs^9,21,22^, CT scans^23^ or ultrasound^24^. It has not been identified in the known literature as a proposal without the use of medical images that meets the requirements of a screening device^25^ for osteoporosis, i.e., one that is more accessible, less invasive, cheaper, faster, and that has been tested on humans.

In terms of effectiveness (fractures prevented), bone mineral density screening is a great option for osteoporosis diagnose^26^. Once the patient has an early diagnosis, he or she may be conducted for physical activity, supplemental vitamin D, and calcium, and in situations of greater risk, pharmacological treatment may be indicated. On the other hand, with Osseus, healthy individuals can be prevented from being referred for DXA, which means cost savings with displacement (for situations in cities that do not have DXA) and exams.

Predicting that a patient has low BMD without having it (false positive) is not a big problem since the prescribed treatment is a healthier lifestyle. Otherwise, classifying a patient with low BMD as healthy (false negative) may cause more serious health issues. Therefore, efforts should be made to minimize false negative errors in future work. A pattern observed during data collection was the use of calcium and/or vitamin D in patients diagnosed with osteoporosis or low BMD, 220 (43.6%). Patients younger than 50 years old, 46 (9.1%) were using corticosteroids, which draws attention to a tendency towards referral of young patients with autoimmune diseases with prolonged use of corticosteroids, such as Lupus, rheumatoid arthritis, pituitary syndromes and hypogonadism, to name a few. With the greater access of the young population to a screening exam, treatment can be started early, avoiding fractures, surgeries, and hospitalization.

Osseus proved to be a good screening tool in regions with limited resources where DXA is not available, and even as an alternative to the gold standard equipment since it does not use ionizing radiation and has no restrictions on use, i.e., the patient can perform the procedure as many times as necessary. In this way, Osseus can act as point-of-care equipment with high recall, even as a public policy in Primary Health Care for screening people with a predisposition to osteoporosis, a silent disease that the sooner treatment is started, the greater the chance to avoid complications such as fractures.

This study had some limitations: the fact that the dataset was not homogeneous or balanced since the patients that appeared in the dataset had already performed a DXA exam, so they were already sick in most of the cases. This imbalance can affect the prediction performance of the algorithm, which is more accurate at identifying sick patients than healthy ones. In future works, we intend to collect a balanced dataset, with a greater number of subjects under the age of 50 and males. Another point to be considered is that the risk factors were self-reported, so the data could be biased due to forgetfulness or omission. In addition, the sample was divided into two classes since it is a screening, and therefore individuals with low bone mineral density already have an indication for DXA.

## Conclusion

The findings of this study show that the RF model proved to be an adequate model for predicting osteoporosis risk, with few hyperparameters and allowing the importance of variables. A device based on microwaves (Osseus) was proposed to recommend whether the patient needs a densitometry exam. Osseus has shown high potential to contribute to the diagnosis of osteoporosis, reduce the amount of unnecessary DXA tests, reduce the risk of fractures by identifying patients early, and improve the patient's perception of this risk so that it is possible to change their lifestyle.

Osseus is an important tool that can be used as an instrument of innovation in health. It can be applied in telehealth to improve the process of medical screening and qualification for the process of regulating access to health services. One of the ways to qualify this process is through the adoption of rapid tests, which can be confirmed through telediagnosis exams for osteometabolic diseases. The incorporation of Osseus in Health Systems, mainly in Primary Health Care (PHC), focuses on aspects related to the expansion of timely access to health services and their rationalization. It positions PHC as the ordering of care and patient-centered. Thus, it is more resolute and assertive in public health.

This study demonstrates how useful a tool like Osseus can be in developing countries based on an experience with applied research in the unified health system of Brazil. It reinforces that strengthening PHC and improving the process of regulating access to health services means reducing inequalities and promoting equity and social justice. Allowing PHC patients access to a rapid test for osteoporosis investigation implies reducing the waiting time for the exam in high complexity, as the health system will only regulate patients who truly require a DXA exam. Therefore, the use of technologies such as Osseus not only promotes a process of digital transformation in health but also directly impacts the rational use of resources spent on public health.

## Materials and methods

### Study design and patient selection

This is a cross-sectional, evaluative study developed in the period from July 2021 and June 2022 at the University Hospital Onofre Lopes of Federal University of Rio Grande do Norte (HUOL) which has 237 beds, 19 of which are in an adult ICU and 5 in a pediatric ICU. The experimental protocol was approved by the Research Ethics Committee of the Federal University of Rio Grande do Norte, Brazil, through CAAE-No. 39675020.0.0000.5292/2020, and in accordance with the Helsinki Accords (as amended in 2004). The calculation of the sample size was determined by the following equation:

$$n = \frac{{z^{2} (P\left( {1 - P} \right)}}{{D^{2} }}$$

where: *P* = proportion expected to test positive for osteoporosis (24%)^27^, D = half-range of the confidence interval (maximum acceptable error of plus or minus 0.04). Z = 1.96 (for α = 0.05 and 95%CI).

The proportion *P* = 24%^27^ used reflects the expectation of a positive osteoporosis test for the general population living in the north and northeast regions of Brazil. The sample ensured an alpha probability below 0.05, specifically 0.0449. For the analysis of males or females, a power of 99% was guaranteed. Considering D = 0.04 and Z = 1.96, the resulting size was 438, which represents the minimum sample size demanded to validate this study. However, after adding 15% of losses, the final value of the sample was 504 patients. Patients who underwent densitometry at the HUOL and met the inclusion criteria established below were eligible to participate in the study on an individual and voluntary basis. The study inclusion criteria were: male or female adults with a prescription for a bone densitometry examination on DXA equipment and signed informed consent by the research participant. Exclusion criteria were defined as: age under 18 years and patients who have an amputated middle finger or one that is too curved, making collection impossible. The first stage of data acquisition consisted of filling out a form with data from the patient's medical records, such as life habits that are risk factors for the disease^28^, which included 21 variables (The complete dataset presented in this study is available in Zenodo at doi.org/10.5281/zenodo.7779063). Then, anthropometric measurements (width, height, and length of the medial phalanx) of the middle finger of the patient's non-dominant hand were taken with a caliper. Afterward, the measurement of the attenuation of the signal emitted without any barrier between the antennas was carried out. This point (frequency of the injected signal) with the highest received power serves as a reference or calibration of the equipment. Finally, the attenuation measurement was performed with the patient's medial phalanx positioned between the antennas. The Osseus measurement has been validated using a network vector analyzer (VNA), Agilent Technologies model E5071C, and a Spectrum Analyzer, Rohde & Schwarz model FSH8, that aid in calibrating the antennas and the attenuation process. The equipment uncertainty is 0.01 mV, which gives a very small error of up to 1%, i.e. the measurement is precise to two decimal points regarding the voltage value measured at the output of the power detector. Concerning the Osseus shielding, it is capable of attenuating external signals by more than 40 dB^29^. At the end of each collection, the result of the DXA exam (GE Lunar DPX Pro) was noted on the form. The total duration of the collection protocol lasted approximately 20 min.

### Dataset preparation

Pre-processing was carried out by searching for calibration and reading values in samples that were out of standard (outliers) in relation to the others, such as, for example, a negative difference in readings or a calibration 50% below the value found in all the others. The medical request defines the site where the DXA will be performed on the patient, which can be spine, femur, forearm, or entire body. The standard deviation values (T-score or Z-score) reported as a result by the machine were recorded in Osseus, and the report was calculated based on the worst standard deviation recorded. The fields of body mass index (BMI) and percentage of attenuation (calibration value minus the reading value divided by the calibration) were also calculated. After these steps, it was possible to calculate the patient's report using the T-score and Z-score tables according to the patient's profile, that is, menopausal women and people over 50 who had a t-score equal to or below − 1 received a report “sick”. The same report was given to samples that were under 50 years of age and that were not menopausal women but that received a Z-score equal to or below − 2. All other samples received a report equal to “healthy” in their records. The score mean and range on each category is detailed in supplementary Table 3.

As the sample is unbalanced and has 395 data from the sick class and 110 from the healthy class, the Synthetic Minority Over-Sampling Technique (SMOTE) method was used, a type of data augmentation for the minority class, in this case the healthy class. This approach involves duplicating examples in the minority class, although these examples don’t add any new information to the model. Instead, new examples can be synthesized from the existing examples. After applying SMOTE, the dataset had 790 examples (395 sick and 395 healthy). Then, 80% of this dataset was randomly selected for the training set and 20% for the test set. Thus, the training set was divided into 5 folds to carry out cross validation with all the classifiers presented before. A seed of 40 was fixed to ensure study reproducibility.

### Model building

The model training aimed to obtain a binary classification of patients; thus, the trained model must assign a label (healthy or sick) for each input (i.e., patient features). Classifier algorithms use labeled data and statistical methods to produce predictions about data input classifications^30^. A total of 19 algorithms that were indicated for this application were tested (Table 2). In general, ensemble models are based on combining several basic algorithms to build a robust model that outperforms any single model. In particular, Random Forests (RF) is built based on combining decision trees and is considered a powerful and versatile machine learning algorithm^31^. Among the advantages of RF:

- Provides an estimate of the importance of the variables^32^;
- They are known to be high performers;
- RF has been used in various applications in bioinformatics^33^, medicine^34^ and public health^35^.

Deep Learning (DL) algorithms are also high-performing and very popular. For the case of this work, we consider that DL models are not a good solution because they demand much more data than we had available; otherwise, these models can easily overfit.

### Model evaluation

With the hyperparameters tuned (Table 3) in the best model, the final test was performed to obtain the performance metrics^36^. The best possible combination of hyperparameters for the RF model consisted of 110 trees, 7 samples as a minimum requirement for splitting the nodes, 6 samples as a minimum for a leaf node, a maximum depth of 8, 7 as the number of variables in each split, and Gini as a criterion for measuring the quality of a split.

**Table 3:** Parameter search in the analysis of randomized search RF.

Parameter	Range	Best result
n_estimator (number of trees in the forest)	[11 uniformly defined numbers between 50 and 200]	110
Min_samples_split (minimum amount of data in a node before the node is split)	[4–8]	7
Min_samples_leaf (minimum number of leaves in a node)	[3–7]	6
max_depth (maximum depth of trees)	[3–9]	8
Max_features (number of variables in each split)	[1 to 21]	7
Criterion	['gini', 'entropy', 'log_loss']	Gini

The confusion matrix (Table 4), computed by the python scikit-learn library, was considered. In addition, the area under the curve (AUC) was also calculated.

**Table 4:** Confusion matrix model used in the study.

	Predictive healthy	Predictive sick	Total
Real healthy	TN	FP	TN + FP
Real sick	FN	TP	FN + TP
Total	TN + FN	FP + TP	TN + FP + FN + TP

*TN* True Negative, *FP* False Positive, *FN* False Negative, *TP* True Positive.

Other metrics used are explained below.

- True Negative Rate (TNR) or specificity is the probability of a negative result in healthy individuals: TN/(FP + TN).
- False Positive Rate (FPR) is the probability of false alarm: FN/(FN + TP).
- False Negative Rate (FNR) is the miss rate: FP/(FP + TN).
- True Positive Rate (TPR) or recall or sensibility is the probability of a positive result in patients: TP/(TP + FN).
- Positive Predictive Value (PPV) or precision is the probability of the presence of the disease when the test is positive: TP/(TP + FP).
- Negative Predictive Value (NPV) is the probability of absence of disease when the test is negative: TN/(FN + TN).
- Accuracy is the probability of the test providing correct results, that is, being positive in patients and negative in healthy ones: (TN + TP)/(TN + FP + FN + TP).
- F1-score is the harmonic mean of the precision and the recall: 2TP/(2TP + FP + FN).

### Supplementary Information

Supplementary Information.

## Data Availability

The complete dataset and data dictionary presented in this study is available in Zenodo at doi.org/10.5281/zenodo.7779063.
