# Supplementary material for: Osteoporosis screening using machine learning and electromagnetic waves
Source: Sci Rep. 2023 Aug 8;13:12865. doi: 10.1038/s41598-023-40104-w (PMC10409756; doi:10.1038/s41598-023-40104-w)
Supplement: Supplementary file 1 — Supplementary Information. [file 41598_2023_40104_MOESM1_ESM.pdf]

Supplementary Information

Osteoporosis Screening using Machine Learning and Electromagnetic Waves

Gabriela A. Albuquerque, Dionísio D. A. de Carvalho, Agnaldo S. Cruz, João Paulo Q. Santos, Guilherme M. Machado, Ignácio S. Gendriz, Felipe R. S. Fernandes, Ingridy M. P. Barbalho, Marquiony M. Santos, César A. D. Teixeira, Jorge M. O. Henriques, Paulo Gil, Adrião D. D. Neto, Antonio L. P. S. Campos, Josivan G. Lima, Jailton C. Paiva, Antonio H. F. Morais, Thaisa Santos Lima, Ricardo A. M. Valentim

The dataset and data dictionary is available on DOI: 10.5281/zenodo.7779063

Table 1 - Data dictionary

Name	Description	Values/Types
Gender	Gender of the patient	Text<br>Options: male and female
Age	Age of the patient	integer
Weight	Weight (kg) of the patient	integer
Height	Height (cm) of the patient	integer
Race	Race of the patient	Text<br>Options: white, black, brown
SD	Standard deviation of the bone density reading pointed out by DXA (T-score or Z-score)	Float
DXA class	DXA reported result	Text<br>Options: normal, low bone mineral density, osteoporosis
Alcohol	Patient consumes alcoholic beverages	Text<br>Option: yes or no

Smoking	Patient smokes or has smoked	Text<br>Option: yes or no
Activity	Patient practices physical activity	Text<br>Option: yes or no
Milk	Patient consumes milk	Text<br>Option: yes or no
Calcium	Patient takes calcium supplements	Text<br>Option: yes or no
Vitamin D	Patient exposes himself to the sun or takes vitamin D supplements	Text<br>Option: yes or no
Fall	Patient suffered a fracture due to a fall	Text<br>Option: yes or no
Parents osteoporosis	Parents has been diagnosed with osteoporosis or has broken a bone after a minor fall	Text<br>Option: yes or no
Parents curved	Parents become hunched over	Text<br>Option: yes or no
Corticosteroids	Patient took corticosteroids for more than 3 months	Text<br>Option: yes or no
Arthritis	Patient was diagnosed with rheumatoid arthritis	Text<br>Option: yes or no
Diseases	Hyperthyroidism, overactive parathyroid glands, diabetes, or gastrointestinal disorders such as Crohn's disease or celiac disease	Text<br>Option: yes or no
Menopause	Patient is in menopause	Text<br>Option: yes or no
Medial_length	Length of the medial phalanx in mm	Integer
Medial_width	Width of the medial phalanx in mm	Integer
Medial_height	Height of the medial phalanx in mm	Integer
Calibration	Osseus signal strength with no obstacle between the antennas	Float
Attenuation	Osseus signal strength with obstacle between antennas	Float

Table 2 - The individual features' importance

Feature name	Individual importance
'age'	0,227401422
'bmi'	0,134514324
'attenuation'	0,090009995
'menopause'	0,082911314
'medial_length'	0,070223803
'calcium'	0,059159597
'medial_width'	0,044219638
'smoking'	0,04115462
'medial_height'	0,044854411
'vitamin_d'	0,037013363
'ethnicity'	0,030213327
'fall'	0,023385329
'parents_osteoporosis'	0,022814332
'disease'	0,021100977
'milk'	0,015772743
'gender'	0,014214909
'activity'	0,012339511
'corticosteroid'	0,012697214
'alcohol'	0,010059062
'arthritis'	0,004432955
'parents_curved'	0,001507154

Table 3 - score mean and range on each category.

**Score**	**Mean**	**Range of values**
**T-Score (455 samples):**		
Healthy (76):	-0.31	[-0.9, 1.9]
Sick (379):	-2.44	[-5.4, -1.0]
**Z-Score (50 samples):**		
Healthy (34):	-0.77	[-2.0, 2.7]
Sick (16):	-2.79	[-4.3, -2.1]

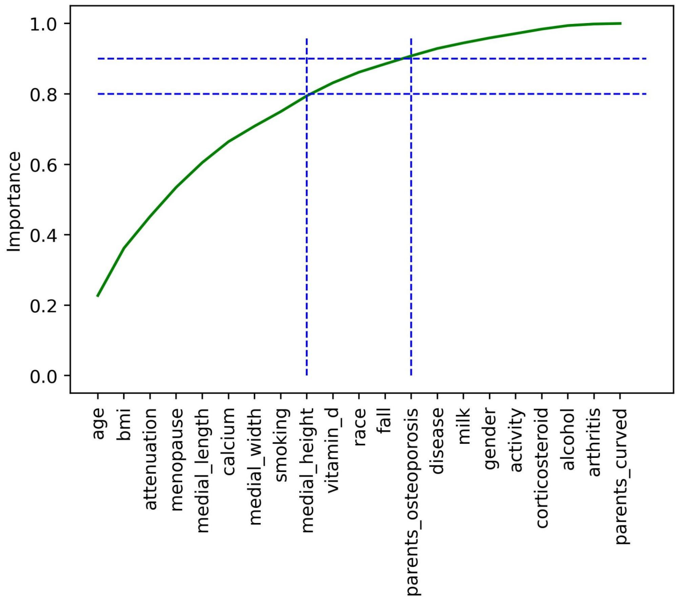

Fig. 1.a. Cumulative importance of features

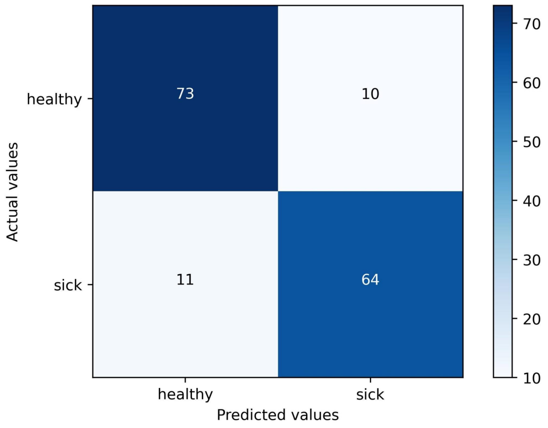

Fig 1.b. All features

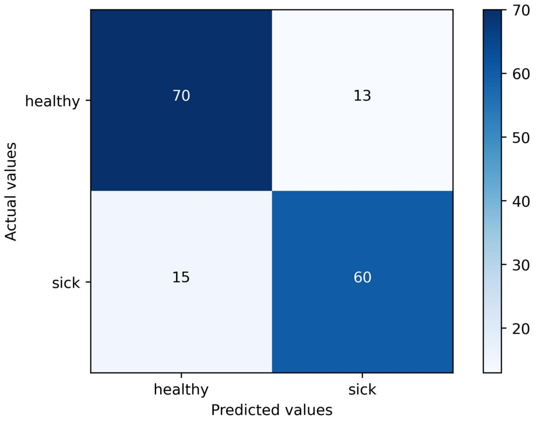

Fig 1.c. Without Osseus signal

Shapiro-wilk results for normal distribution

Shapiro-wilk (all features): 0.43

Comparison all features vs. without Osseus

Shapiro-wilk (without Osseus signal): 0.66

T-test: 0.75

p-value: 0.46

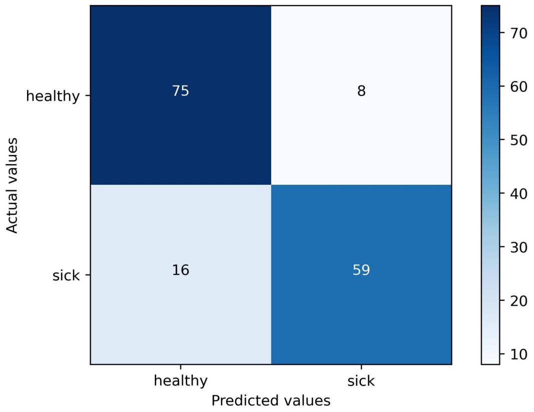

Fig 1.d. Without Age

Comparison all features vs. without age

Shapiro-wilk (without age): 0.30

T-test: 1.70,

p-value: 0.10
